# The ClpS-like N-domain is essential for the functioning of Ubr11, an N-recognin in *Schizosaccharomyces pombe*

**DOI:** 10.1186/2193-1801-3-257

**Published:** 2014-05-20

**Authors:** Kenji Kitamura

**Affiliations:** Center for Gene Science, Hiroshima University, 1-4-2 Kagamiyama, Higashi-Hiroshima, 739-8527 Japan

**Keywords:** Ubr protein, Ubiquitin ligase, N-end rule pathway, N-recognin, Oligopeptide transporter, Yeast

## Abstract

Several Ubr ubiquitin ligases recognize the N-terminal amino acid of substrate proteins and promote their degradation via the Arg/N-end rule pathway. The primary destabilizing N-terminal amino acids in yeast are classified into type 1 (Arg, Lys, and His) and type 2 (Phe, Trp, Tyr, Leu, Ile, and Met-Ф) residues. The type 1 and type 2 residues bind to the UBR box and the ClpS/N-domain, respectively, in canonical Ubr ubiquitin ligases that act as N-recognins. In this study, the requirement for type 1 and type 2 amino acid recognition by *Schizosaccharomyces pombe* Ubr11 was examined *in vivo*. Consistent with the results of previous studies, the *ubr11*∆ null mutant was found to be defective in oligopeptide uptake and resistant to ergosterol synthesis inhibitors. Furthermore, the *ubr11*∆ mutant was also less sensitive to some protein synthesis inhibitors. A *ubr11* ClpS/N-domain mutant, which retained ubiquitin ligase activity but could not recognize type 2 amino acids, phenocopied all known defects of the *ubr11*∆ mutant. However, the recognition of type 1 residues by Ubr11 was not required for its functioning, and no severe physiological abnormalities were observed in a *ubr11* mutant defective in the recognition of type 1 residues. These results reinforce the fundamental importance of the ClpS/N-domain for the functioning of the N-recognin, Ubr11.

**Electronic supplementary material:**

The online version of this article (doi:10.1186/2193-1801-3-257) contains supplementary material, which is available to authorized users.

## Background

The N-end rule pathway is responsible for the ubiquitination of substrate proteins that harbor a destabilizing amino acid at their N-terminus. The destabilizing amino acid is an essential component of a degradation signal known as the N-degron. A Ubr ubiquitin ligase, acting as an N-recognin, binds to the N-terminal residue of the N-degron and ubiquitinates the substrate protein, thus targeting it for degradation by the proteasome (Tasaki et al. 2012; Varshavsky 2011). Two independent pathways, the Arg/N-end rule and the acetylated N-end rule, have been described for this process. Ubr ubiquitin ligases function only in the Arg/N-end rule pathway.

Arg/N-end rule N-recognin mutants of the yeasts, *Saccharomyces cerevisiae* (*ubr1*) and *Schizosaccharomyces pombe* (*ubr11*), grow almost normally. However, because the Ubr proteins are required for the expression of oligopeptide transporters, both mutants are defective in the uptake of extracellular oligopeptides (Byrd et al. 1998; Kitamura et al. 2012). The regulation of peptide uptake has been well characterized in *S. cerevisiae* (Turner et al. 2000; Du et al. 2002; Xia et al. 2008a, b; Varshavsky 2011). After Ubr1 ubiquitinates and promotes the degradation of the transcriptional repressor, Cup9, the expression of the peptide transporter gene (*PTR2*) is upregulated, and the cell becomes competent for peptide uptake. The binding of the intracellular N-end rule dipeptides to Ubr1 facilitates the recognition and ubiquitination of Cup9 by Ubr1, and further accelerates peptide uptake. Similarly, Ubr11 in *S. pombe* is essential for the expression of the oligopeptide transporter genes, *ptr2* and *isp4* (Kitamura et al. 2012). However, no Cup9 homolog has been found in the *S. pombe* genome, and the Ubr11 substrate that represses peptide uptake in *S. pombe* has not been identified. In the opportunistic pathogen, *Candida albicans*, Ubr1 controls hyphal initiation through the proteolysis of Cup9. However, it is not known whether the degradation of Cup9 depends on the type of residue present at its N-terminus (Lu et al. 2014). Similarly, the Ubr1-related protein, Ubl1, in the plant pathogen *Fusarium* is necessary for virulence against wheat and maize; however, the relevant substrate proteins are unknown (Ridenour et al. 2013).

The classical primary destabilizing amino acid in the N-degrons of the Arg/N-end rule pathway in yeast is either a basic residue (type 1: Arg, Lys, or His) or a bulky hydrophobic residue (type 2: Leu, Ile, Trp, Phe, or Tyr) (Varshavsky 2011; Tasaki et al. 2012). An unacetylated N-terminal methionine followed by a hydrophobic residue (designated Met-Ф also functions as a type 2 N-degron (Kim et al. 2014). Different regions within the canonical Ubr N-recognin proteins bind to type 1 and type 2 amino acids (Xia et al. 2008b; Tasaki et al. 2009). Type 2 N-terminal amino acids bind to the N-domain, which is homologous to the bacterial N-recognin, ClpS (Lupas and Koretke 2003; Erbse et al. 2006; Tasaki et al. 2009). Similar to the eukaryotic N-end rule pathway, ClpS is responsible for the recognition of the same N-terminal type 2 amino acids (except Ile) in the bacterial N-end rule pathway (Erbse et al. 2006; Dougan et al. 2010). In contrast, type 1 N-terminal residues are recognized by the eukaryote-specific UBR domain. In our previous studies, we investigated the roles of these domains and demonstrated the importance of type 2 N-terminal amino acid recognition in peptide uptake, by mutating Ubr11 in *S. pombe* (Kitamura and Fujiwara 2013). However, how the recognition of type 1 residues affects the *in vivo* function of Ubr11 was not characterized.

In the present study, a Ubr11 mutant that was defective only in the recognition of type 1 N-terminal amino acids was engineered, and the effects of the mutation were compared with those in a Ubr11 ClpS/N-domain mutant defective in the recognition of type 2 residues. Importantly, it was found that the recognition of type 2 residues by the ClpS/N-domain was essential, but the recognition of type 1 residues by the UBR domain was dispensable for almost all Ubr11 functions. These results contribute to our understanding of the structure-function relationship in canonical Ubr ubiquitin ligases.

## Results

### Analysis of a *ubr11*mutant defective in type 1 amino acid recognition

**Figure 1:**
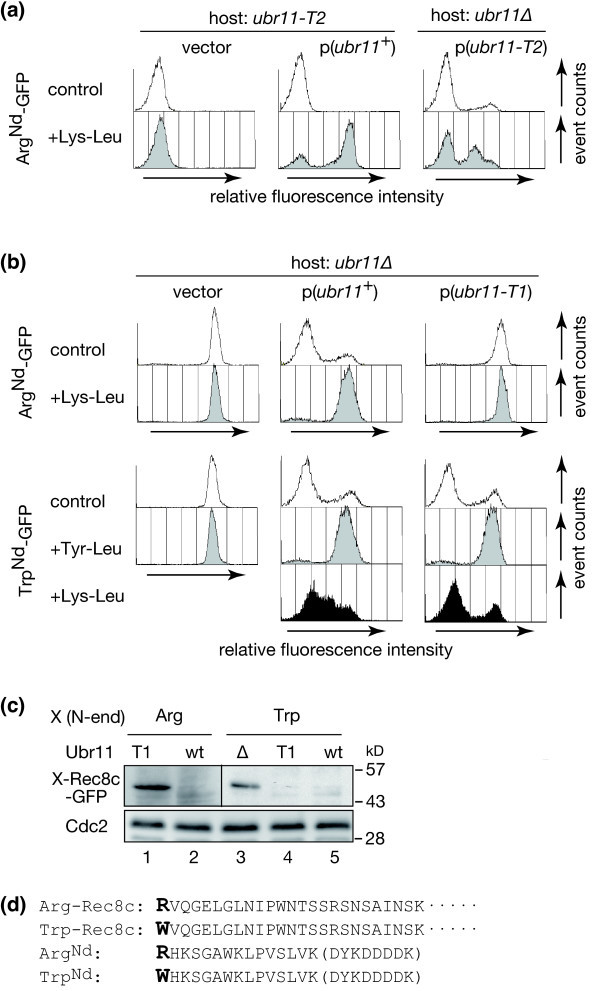
**Characterization of** ***ubr11*** **mutants specifically defective in the recognition of N-terminal type 1 or type 2 residues.** **(a and** **b)** Each strain was cultured with or without the indicated dipeptide for 4 h. The relative fluorescence intensities of Arg^Nd^-GFP and Trp^Nd^-GFP, which are type 1 and type 2 N-end rule substrates, respectively, were measured by flow cytometry. **(a)** The effect of Lys-Leu dipeptides was severely compromised in the *ubr11-T2* mutant. *ubr11-T2*, which has type 2 N-terminal residue-specific recognition defect (left and middle), or the *ubr11*∆ (right) strains, which express Arg^Nd^-GFP, were transformed with the indicated plasmid, and monitored for changes in the fluorescence intensity of Arg^Nd^-GFP. **(b)** Ubr11-T1 was specifically defective in the recognition of type 1 N-end residues. Each strain was examined as described in **(a)**. **(c)** The type 1 N-degron sequence in Rec8 did not promote Rec8c degradation in the *ubr11-T1* strain. The X-Rec8c-GFP was expressed from the *nmt* promoter. The steady state levels of the type 1 substrate, Arg-Rec8c-GFP (lanes 1 and 2), and the type 2 substrate, Trp-Rec8c-GFP (lanes 3–5), in the indicated strains were examined by immunoblotting with anti-GFP antibody. Cdc2: loading control. **(d)** Amino acid sequences of the N-degrons used in this study. The DYKDDDDK sequence shown in parenthesis is a FLAG tag epitope that was inserted before the GFP protein.

The residues of bacterial ClpS that interact with the hydrophobic N-degrons were identified in earlier studies (Wang et al. 2008; Román-Hernández et al. 2009; Schuenemann et al. 2009). We previously characterized a yeast strain that harbored a mutation within the conserved ClpS/N-domain in *S. pombe* Ubr11. This *ubr11-m3* mutant (D251N, H254Y), which was renamed *ubr11-T2* in this study (Additional file 1: Figure S1) because of its ‘type 2-defective’ nature, lacked dipeptide uptake because of its inability to express the Ptr2 peptide transporter (Kitamura and Fujiwara 2013). In the *ubr11-T2* mutant, which was unable to recognize type 2 residues, the fluorescence intensity of the type 1 model substrate, Arg^Nd^-fused green fluorescent protein (Arg^Nd^-GFP), was low, similar to that in wild type Ubr11-expressing cells (control profiles in Figure 1a). We previously demonstrated that the fluorescence intensity reflects the protein amount of an N-degron, Xaa^Nd^ bearing-GFP, and that a low GFP level correlates with an intrinsic instability of GFP, because of its N-end rule-dependent proteolysis (Kitamura and Fujiwara 2013). Because the N-end rule substrate and the N-end rule dipeptide compete for the same binding site within Ubr11, proteolysis via the N-end rule pathway is inhibited by dipeptides that bear the same type of N-terminal amino acid as the substrate. In contrast to the wild type Ubr11-expressing cells, degradation was not inhibited by exogenous Lys-Leu type 1 dipeptides in the *ubr11-T2* mutant (Figure 1a, vector) because it was defective in the expression of the Ptr2 dipeptide transporter (Kitamura and Fujiwara 2013). However, when a multicopy plasmid was used to increase the expression of Ubr11-T2, Lys-Leu dipeptides weakly inhibited the degradation of Arg^Nd^-GFP, although not as effectively as in a strain expressing wild type Ubr11 (Figure 1a, right, and b; Additional file 2: Figure S2b). Ubr11’s recognition of Lys-Leu is not affected by the *ubr11-T2* mutation itself (Kitamura and Fujiwara 2013). Therefore, these results indicated that Ubr11-T2 was not completely inactive, but its ability to induce peptide uptake was severely compromised.

**Figure 2:**
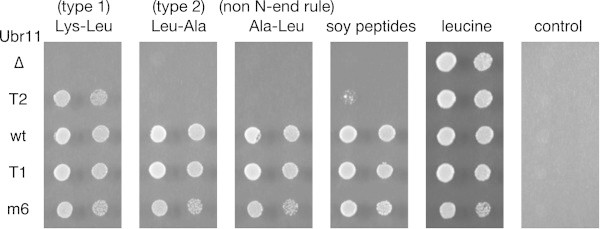
**Uptake of dipeptides by the** ***ubr11*** **mutants.** A host strain (*ubr11*∆ *leu1 ura4*) was transformed with *ura4* ^+^ plasmids encoding Ubr11 mutant or wild type proteins. Their ability to rescue the growth defect of the leucine auxotrophic host strain in the presence of leucine-containing dipeptides was tested. Serially diluted cells were spotted on the medium containing the indicated peptides or leucine. The control medium lacked any source of leucine. The *ubr11-T1* mutation, which impaired recognition of type 1 residues, did not affect the utilization of the dipeptides. In contrast, the type 2-specific *ubr11-T2* mutant was mostly defective.

To complement the above findings and to further investigate the relationship between N-terminal amino acid recognition and peptide uptake, a Ubr11 mutant defective in the recognition of type 1 residues was characterized. The *ubr11-m6* mutant (Asp117Ala; Additional file 1: Figure S1) was tested first because the conserved aspartic acid interacts with the type 1 N-terminal amino acid of substrate proteins (Choi et al. 2010; Matta-Camacho et al. 2010). Further, a corresponding mutation in mouse Ubr1 specifically interferes with its ability to bind type 1 amino acids *in vitro* (Tasaki et al. 2009). However, its functionality *in vivo* was not tested. The mutant Ubr11-m6 protein was expressed in the *ubr11*∆ strain from a multicopy plasmid, and its functionality was monitored. Interestingly, the *S. pombe ubr11-m6* mutant was able to degrade both type 1 and type 2 model substrates (Additional file 2: Figure S2a and b) and induce peptide uptake (Figure 2). Another mutant, *ubr11-T1* (Gly147Arg), was then examined because a mutation of the corresponding residue in *S. cerevisiae* Ubr1 produced a mutant that was defective in targeting type 1 substrates (Xia et al. 2008b). This conserved glycine is also located near the residue critical for type 1 N-end recognition (Additional file 1: Figure S1). The type 1 model substrate, Arg^Nd^-GFP, was highly expressed in the *S. pombe ubr11-T1* mutant (Figure 1b, top right panel; Additional file 2: Figure S2b), as in the control *ubr11*∆ strain (Figure 1b, top left panel, vector; Additional file 2: Figure S2b). In contrast, the fluorescence intensity of the type 2 substrate, Trp^Nd^-GFP, was low and its level increased when the type 2 dipeptide, Tyr-Leu, was added to strains expressing either wild type Ubr11 or Ubr11-T1. Therefore, the ubiquitin ligase activity of Ubr11-T1 was not impaired, and the *ubr11-T1* mutant had a specific defect in type 1 residue recognition, but responded normally to the type 2 dipeptide. For reasons that are still unclear, the fluorescence intensity and protein levels of the type 2 substrate, Trp^Nd^-GFP, were partially increased when type 1 Lys-Leu dipeptides were added to a strain expressing wild type Ubr11 (Figure 1b, left profile shown in black; Additional file 3: Figure S3a and b). However, Lys-Leu dipeptides were not effective in the *ubr11-T1* mutant (Figure 1b), confirming that this mutant had lost the ability to interact with type 1 N-terminal amino acids.

To exclude the possibility that the defect in the *ubr11-T1* mutant was confined to the Arg^Nd^ N-degron, the stability of another N-end rule substrate, bearing a different N-degron, was examined. The C-terminal fragment of Rec8 (Rec8c), which is generated by a separase-mediated cleavage of cohesin, is an endogenous substrate of the Arg/N-end rule pathway in *S. pombe* (Fujiwara et al. 2013). Although the N-degron sequence in Arg-Rec8c is completely different from that in Arg^Nd^ (Figure 1d), Arg-Rec8c-GFP (type 1 substrate), but not Trp-Rec8c-GFP (type 2 substrate), was stabilized in the *ubr11-T1* mutant (Figure 1c). Therefore, it was concluded that the *ubr11-T1* mutant was defective in the recognition of N-terminal type 1 amino acids in general.

### Utilization of dipeptides by ubr11 mutants defective in recognizing type 1 or type 2 residues

Subsequently, the ability of the mutants to support peptide uptake was tested. All proteins were expressed in the *ubr11*∆ host strain from a multicopy plasmid. Consistent with our previous report (Kitamura et al. 2012), *ubr11*∆ cells harboring the empty vector were defective in the uptake of all dipeptides examined (Figure 2). Interestingly, the uptake of all three dipeptides was not affected in the *ubr11-T1* mutant. However, the *ubr11-T2* mutant plasmid failed to rescue the growth when type 2 (Leu-Ala) or non-N-end rule (Ala-Leu) dipeptides were used. Growth could not be recovered even when soy peptides (various soy-derived oligopeptides-enriched mixtures; Kitamura et al. 2012) were used as a source of leucine. Although a *ubr11*∆ strain harboring the multicopy *ubr11*-T2 plasmid was able to utilize type 1 dipeptides (Lys-Leu), a mutant strain, in which the genomic *ubr11*^+^ locus was replaced with the *ubr11-T2* gene, failed to utilize Lys-Leu (Kitamura and Fujiwara 2013). In conclusion, Ubr11 stimulated dipeptide uptake even when it was unable to recognize N-terminal type 1 amino acids. In contrast, recognition of type 2 amino acids had a pivotal role in peptide uptake.

### Resistance of *ubr11*mutants to protein synthesis inhibitors

**Figure 3:**
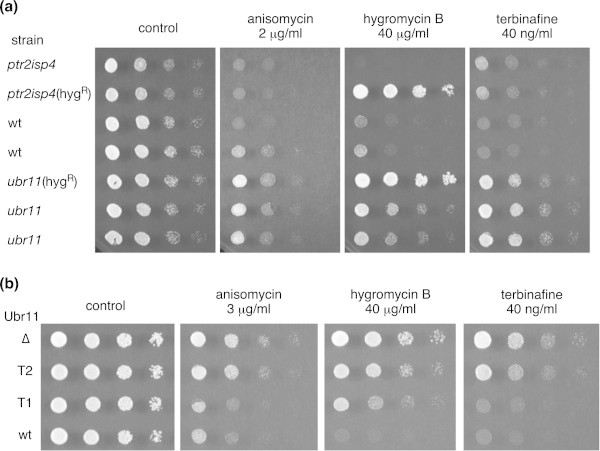
**The** ***ubr11-T2*** **mutant phenocopied** ***ubr11*** **∆ mutant for drug resistance. (a)** Resistance of the *ubr11*∆ mutant to anisomycin and hygromycin B (inhibitors of protein synthesis) and terbinafine (an inhibitor of ergosterol synthesis). Serially diluted cells of each strain were spotted on drug-containing or drug-free (control) medium. Note that the strains shown in the second and fifth rows are intrinsically resistant to hygromycin B because they contain the *isp4*::hphMX6 or *ubr11*::hphMX6 allele. **(b)** The host *ubr11*∆ strain was transformed with plasmids encoding wild type or mutant Ubr11, and the sensitivity of the yeast to the inhibitors was examined as in **(a)**. The *ubr11*∆ and *ubr11-T2* strains showed the same profiles, but the *ubr11-T1* profile resembled that of the wild type strain.

When characterizing the *ubr* mutants, it was observed that the *ubr11*∆ mutant showed weak resistance towards low doses of protein synthesis inhibitors, such as anisomycin and hygromycin B (Figure 3a). The expression of two major oligopeptide transporters, Ptr2 and Isp4, is very low in *ubr11*∆ cells (Kitamura et al. 2012). However, like the wild type strain, the *ptr2*∆ *isp4*∆ double mutant was sensitive to both inhibitors, indicating that the *ubr11*∆ mutant was inherently less sensitive to inhibition of peptide synthesis, and that Ptr2 and Isp4 were unrelated to the resistance. Next, the effect of type 1 and type 2 recognition defects on the cells’ sensitivity to protein synthesis inhibitors was tested. Only the *ubr11-T2* mutant, but not *ubr11-T1*, was resistant to anisomycin (Figure 3b). Both the *ubr11-T1* and the *ubr11-T2* mutants were resistant to hygromycin B at 40 μg/mL, though the effect on Ubr11-T2 was more pronounced. These results suggested that an impairment in the functionality of the ClpS/N-domain affected the sensitivity towards these protein synthesis inhibitors.

### Mutation in the ubr11 ClpS/N-domain mitigated the growth inhibitory effects of terbinafine

A *ubr11*∆ mutant was identified in a genome-wide screen for mutants that were resistant to terbinafine and clotrimazole, both of which inhibit different steps of the ergosterol synthetic pathway (Fang et al. 2012). Although the reason for the resistance remains unclear, the resistance to terbinafine was confirmed using an independently prepared *ubr11*∆ strain; it was established that both Ptr2 and Isp4 were not responsible for the resistance (Figure 3a). Interestingly, the *ubr11-T2* ClpS/N-domain mutant was also resistant to terbinafine, whereas cells expressing Ubr11-T1 or the wild type protein were sensitive (Figure 3b), suggesting that the integrity of the ClpS/N-domain is also important for the response towards terbinafine.

## Discussion

In this study, the relationship between the recognition of N-terminal residues in the Arg/N-end rule pathway and the *in vivo* function of the N-recognin, Ubr11, in *S. pombe* was examined. The Arg/N-end rule pathway in yeast plays an important role in the regulation of extracellular oligopeptide uptake by promoting the expression of peptide transporters (Varshavsky 2011; Kitamura et al. 2012). In *S. cerevisiae*, the Ubr1-dependent proteolysis of the transcriptional repressor, Cup9, by the Arg/N-end rule pathway is essential for peptide uptake (Turner et al. 2000; Du et al. 2002; Xia et al. 2008a). Curiously, Cup9 is neither a type 1 nor a type 2 N-end rule substrate (Xia et al. 2008b). The binding of N-end rule dipeptides to the N-recognin, Ubr1, increases the degradation of Cup9, thus further stimulating peptide uptake (Turner et al. 2000). Interestingly, the *S. pombe* genome does not encode a Cup9 homolog. In this study, in *S. pombe*, the recognition of N-terminal type 1 amino acids was found to be dispensable, whereas the recognition of type 2 amino acids was found to be critical for the *in vivo* function of Ubr11. Based on the results of this study, we can assume that the N-end rule substrate required for peptide uptake in *S. pombe* may be a type 2 substrate protein. However, the identity of this protein remains unknown. Alternatively, the ClpS/N-domain of Ubr11 may regulate peptide uptake by recognizing an intracellular type 2 oligopeptide itself, rather than a type 2 substrate protein.

To date, several regulators of cellular physiology, including transcription factors that regulate hypoxic responses, caspase-generated pro-apoptotic protein fragments, neurodegeneration-associated protein fragments, proteolytic fragment of BRCA1 (breast cancer susceptibility type 1 protein), and PINK1 (a Parkinson disease-related mitochondrial protein) have been identified as substrates of the Arg/N-end rule pathway in plants and mammals (Gibbs et al. 2011; Licausi et al. 2011; Piatkov et al. 2012; Xu et al. 2012; Brower et al. 2013; Yamamoto and Youle 2013). However, these proteins are not conserved in yeast. In *S. cerevisiae* and *S. pombe*, other than the type 2 Met-Ф degron-bearing proteins, whose N-termini are inherently acetylated (Kim et al. 2014), only the C-terminal fragments of the mitotic cohesin subunit, Scc1, and its meiotic counterpart, Rec8, have been identified as Arg/N-end rule substrates whose degradation depends on the N-terminal residue (Rao et al. 2001; Fujiwara et al. 2013). The C-terminal fragments of Scc1 and Rec8, generated by a separase-mediated cleavage during mitosis and meiosis, respectively, bear a type 1 N-end residue. Inhibiting the degradation of the Scc1 and Rec8 fragments is not deleterious unless the proteins are strongly overexpressed from an ectopic promoter (Rao et al. 2001; Fujiwara et al. 2013). Taken together with our finding that a *ubr11-T1* mutation does not result in any apparent defects and that the *ubr11*∆ mutant has no adverse phenotypes in meiosis (Fujiwara et al. 2013), these observations suggest that there may be no essential type 1 N-end rule substrates that need to be degraded in *S. pombe*, at least in an unperturbed condition. It is still possible that the degradation of an unidentified type 1 substrate is required for survival in a specific condition. However, such drugs or conditions, under which the *ubr11* mutation results in deleterious effects in *S. pombe*, have not been encountered. Apparently, the degradation of type 1 substrates by the Arg/N-end rule pathway may have gained significance in higher eukaryotes during the course of evolution.

Type 1 N-degrons appear to induce proteolysis more potently than type 2 N-degrons in *S. pombe* (Kitamura and Fujiwara 2013; Fujiwara et al. 2013). However, the recognition of type 2 amino acids seems to play a more important role than the recognition of type 1 residues, as demonstrated in this study. Intriguingly, among the canonical Ubr proteins, the ClpS/N-domain, which recognizes type 2 amino acids, is conserved only in genuine N-recognins (Tasaki et al. 2012). The N-domain sequence in Ubr proteins was originally identified on the basis of its homology to a small bacterial N-recognin protein, ClpS (Lupas and Koretke 2003; Erbse et al. 2006; Tasaki et al. 2009). By incorporating the ClpS-like N-domain, canonical Ubr N-recognins in eukaryotes acquired a specialized role for the recognition of N-end residue in the Arg/N-end rule pathway and, simultaneously, a means to regulate peptide uptake. The binding region for type 1 residues is found within the eukaryote-specific UBR domain that characterizes all Ubr proteins, including the non-N-recognins and the non-canonical Ubr proteins (mammalian Ubr3–Ubr7, *S. pombe* Ubr1, and *S. cerevisiae* Ubr2) (Varshavsky 2011; Tasaki et al. 2012). Previous studies have shown that the degradation of type 2 N-end rule substrates is stimulated in the presence of type 1 dipeptides (Reiss et al. 1988; Gonda et al. 1989; Baker and Varshavsky 1991; Kwon et al. 2001). Considering this, an unexpected observation in this study was that exogenous type 1 dipeptides increased the amount of type 2 substrate. In the case of Trp^Nd^-GFP, an increase was observed for at least two different type 1 dipeptides, Lys-Leu and Arg-Phe (Figure 1b, Additional file 3: Figure S3). In contrast, the type 2 dipeptide, Tyr-Leu, did not affect the levels of Arg^Nd^-GFP, a type 1 substrate (Kitamura and Fujiwara 2013). Although the molecular mechanisms underlying this phenomenon need to be elucidated in future studies, the current data indicate that the recognition of type 2 substrates by N-recognins is strongly influenced by the presence of type 1 dipeptides in a complex manner.

Ubr ubiquitin ligases are necessary for protein quality control (Eisele and Wolf 2008; Heck et al. 2010; Nillegoda et al. 2010; Prasad et al. 2010; Khosrow-Khavar et al. 2012; Theodoraki et al. 2012: Stolz et al. 2013; Summers et al. 2013; Kriegenburg et al. 2014). In both *S. cerevisiae* and *S. pombe*, a mutation in the Ubr N-recognin alleviates growth defects in several temperature-sensitive mutants (Khosrow-Khavar et al. 2012; Kriegenburg et al. 2014). In this study, it was found that the *ubr11*∆ mutant was less sensitive to protein synthesis inhibitors than the wild type strains, suggesting that Ubr11 also contributes to quality control when partially defective proteins that retain some residual function are produced in the presence of low concentrations of protein synthesis inhibitors. Alternatively, substrates of Ubr11 may be required for certain critical cellular processes that are more sensitive to protein level alterations. In either case, inactivation of the Ubr11-dependent proteolysis of substrates alleviates growth inhibition. Interestingly, in *S. cerevisiae,* the quality control of several proteins is mediated by the Arg/N-end rule pathway, in which an unacetylated N-terminal methionine is recognized by the type 2 amino acid-binding site within Ubr1 (Kim et al. 2014). However, other studies have shown that the role of the Ubr proteins in quality control is independent of the N-end rule pathway (Heck et al. 2010; Nillegoda et al. 2010). Whether the Ubr proteins function as N-recognins or act independently of the N-end rule pathway may depend on the substrate protein. As shown in this study, the *ubr11* ClpS/N-domain mutant displayed altered responses to several drugs. Whether these defects involve recognition of substrates with type 2 N-end residues remains to be determined.

## Conclusions

Ubr11 N-recognin in *Schizosaccharomyces pombe* is a conserved ubiquitin ligase, which is essential for the degradation of substrate proteins harboring destabilizing N-terminal amino acids (N-degron) via the Arg/N-end rule pathway. The N-domain in Ubr11, which is homologous to the bacterial ClpS, recognizes bulky hydrophobic amino acids at the N-terminus (type 2 N-degron). This report described a ClpS/N-domain mutant, which did not recognize type 2 N-end residues but retained ubiquitin ligase activity towards type 1 substrates, phenocopied the *ubr11*∆ null mutant. Specifically, the ClpS/N-domain mutant was resistant to inhibitors of ergosterol synthesis (terbinafine) and protein synthesis (hygromycin B and anisomycin). These findings indicate that the ClpS/N-domain has a general role in all cellular functions of the Ubr11 protein, in addition to its known role as a recognition site for N-degron.

## Materials and methods

### Yeast strains and culture conditions

The yeast strains used in this study are listed in Additional file 4: Table S1. Rich complete medium (YES) and synthetic minimal medium (EMM2) were used for cell culture. These media and other general yeast methods have been described previously (Forsburg and Rhind 2006). Ammonium chloride (the nitrogen source in EMM2) was replaced with sodium glutamate (0.38% w/v) to evaluate the sensitivity of yeast to anisomycin, hygromycin B, and terbinafine. Dipeptides were purchased from Sigma-Aldrich Japan (Tokyo, Japan), Bachem (Bubendorf, Switzerland), and Kokusan Chemical Co. Ltd. (Tokyo, Japan), and used at 0.2 mM (to support cell growth) or 5 mM (to inhibit proteolysis). Hi-Nute HK soy peptides (provided by Ms. Kitagawa, Fuji Oil, Osaka, Japan) were used at a concentration of 0.1% (w/v).

To monitor proteolysis via the Arg/N-end rule pathway, the GFP-tagged model substrates, Xaa^Nd^-GFP and X-Rec8c-GFP, were used as described previously (Fujiwara et al. 2013; Kitamura and Fujiwara 2013). To express these proteins from the *nmt* promoter, the cells were grown in thiamine-free EMM2 for at least 18 h at 28°C. To inhibit degradation via the Arg/N-end rule pathway, the cells were treated with dipeptides for 3–5 h.

### Plasmids

The *ubr11-m6* (Asp117Ala) and *ubr11-T1* (Gly147Arg) mutants were synthesized as described previously by inverse polymerase chain reaction using the Pk-*ubr11*^+^ template plasmid (Kitamura and Fujiwara 2013). After sequence verification, each *ubr11* gene, including the promoter region, was excised by *Pst*I digestion and inserted into the *Pst*I site of the pDblet vector (Brun et al. 1995). The *ubr11-T2* mutant, which does not recognize type 2 N-terminal residues, is identical to the *ubr11-m3* mutant, which we reported in previous studies (Kitamura and Fujiwara 2013, Additional file 1: Figure S1). However, the mutant was renamed *ubr11-T2* in this study, to emphasize its type-2 residue-specific defect.

### Flow cytometry

The relative fluorescence intensities of Arg^Nd^-GFP and Trp^Nd^-GFP were measured in 10000 live cells by flow cytometry using a FACSCalibur™ flow cytometer (Becton Dickinson, Franklin Lakes, NJ).

### Immunoblotting

Total cellular protein extracts were prepared and used in immunoblotting experiments as described previously (Kitamura et al. 2011). Anti-GFP (GF200; Nacalai Tesque, Kyoto, Japan) and anti-Cdc2 (sc-53; Santa Cruz Biotechnology, Dallas, Texas) were used as the primary antibodies.

## Electronic supplementary material

Additional file 1: Figure S1: Sequence alignments of Ubr boxes and ClpS/N-domains. Residues that are identical and homologous in at least three proteins are shown in red and blue, respectively. Highlighted in black in the *S. pombe* Ubr11 are the residues mutated in this study. The type 1 and type 2 N-terminal amino acids of substrates or oligopeptides interact with the residues marked by asterisks in the Ubr box and ClpS/N-domain, respectively. Sp, *Schizosaccharomyces pombe*; Sc, *Saccharomyces cerevisiae*; Fv, *Fusarium verticillioides*; Ca, *Candida albicans*; Mm, *Mus musculus*; Ec, *Escherichia coli*; Cc, *Caulobacter crescentus*. (PDF 55 KB)

Additional file 2: Figure S2: Functionality of the *ubr11* mutants. **(a)** The *ubr11-m6* mutant was functional. The *ubr11*∆ strains which expressed Arg^Nd^-GFP (left) or Trp^Nd^-GFP (right) were transformed with an autonomously replicating plasmid encoding the Ubr11-m6 protein, and analyzed by flow cytometry. The GFP fluorescence levels were low in untreated control cells due to proteolysis via the N-end rule pathway. After 3 h of incubation in the presence of the dipeptides, the fluorescence intensities increased to a level similar to that in wild type Ubr11 expressing cells (Figure 1b). The peak to the right, with high fluorescence levels of Trp^Nd^-GFP, in the control culture may represent the population that lost the plasmids because of their relatively unstable nature. **(b)** Functionality of the type 1- and type 2-specific *ubr11* mutants. The Arg^Nd^-GFP-expressing *ubr11*∆ strain was transformed with the empty vector (lane 1, ∆) or plasmids directing the expression of each Ubr11 mutant protein (lanes 2–10). The cells were cultured in the presence or absence of Lys-Leu dipeptides for 5 h, and the GFP protein levels were compared by immunoblotting. The same extracts were independently examined for the *ubr11-T2* mutant (lanes 3, 4, 9, and 10). Eight micrograms (lanes 1–8) and three micrograms (lanes 9 and 10) of total cell extracts were used. Cdc2: loading control. (ZIP 662 KB)

Additional file 3: Figure S3: The type 1 dipeptides induced an increase in the Trp^Nd^-GFP type 2 substrate protein levels. **(a)** Three wild type strains expressing Xaa^Nd^-GFP from the same *nmt1* promoter were cultured for 5 h (Trp^Nd^-GFP and Met^Nd^-GFP) or 3 h (Arg^Nd^-GFP) in the presence or absence of the dipeptides indicated. Relative fluorescence intensities in each culture were examined by flow cytometry. Unexpectedly, Trp^Nd^-GFP fluorescence, in addition to Arg^Nd^-GFP fluorescence, increased in response to two different type 1 dipeptides. In contrast, the levels of Met^Nd^-GFP, which is not a substrate of the N-end rule pathway, were not affected by the dipeptides. Only the N-terminal amino acids (Arg, Trp, and Met) are different among these three GFP proteins, indicating that Lys-Leu did not generally affect the GFP levels. **(b)** Total cell extracts were prepared from the same culture that was analyzed in **(a)**, and protein levels of Trp^Nd^-GFP were examined by immunoblotting. (ZIP 536 KB)

Additional file 4: Table S1: *Schizosaccharomyces pombe* strains used in this study. (DOCX 73 KB)
