# Supplementary material for: The ClpS-like N-domain is essential for the functioning of Ubr11, an N-recognin in Schizosaccharomyces pombe
Source: Springerplus. 2014 May 20;3:257. doi: 10.1186/2193-1801-3-257 (PMC4447728; doi:10.1186/2193-1801-3-257)
Supplement: Supplementary file 1 — Additional file 1: Figure S1: Sequence alignments of Ubr boxes and ClpS/N-domains. Residues that are identical and homologous in at least three proteins are shown in red and blue, respectively. Highlighted in black in the S. pombe Ubr11 are the residues mutated in this study. The type 1 and type 2 N-terminal amino acids of substrates or oligopeptides interact with the residues marked by asterisks in the Ubr box and ClpS/N-domain, respectively. Sp, Schizosaccharomyces pombe; Sc, Saccharomyces cerevisiae; Fv, Fusarium verticillioides; Ca, Candida albicans; Mm, Mus musculus; Ec, Escherichia coli; Cc, Caulobacter crescentus. (PDF 55 KB) [file 40064_2014_1547_MOESM1_ESM.pdf]

Ubr box

```
                             m6(D117A)                      T1(G147R)
                             A                              R
SpUbr11   CGHVFRAGEVIYRCKNCGLDNTCVLCAPCFHATNHEGHETHVSISTSY-SGICDCGDPEAWNVDLNC   98-163
ScUbr1    CGRKFKIGEPLYRCHECGCDDTCVLCIHCFNPKDHVNHHVCTDICTEFTSGICDCGDEEAWNSPLHC  123-189
FvUbl1    CGHIFKPGEASYMCRTCGTDETCCLCARCFDSTDHTGHMVRIHISVGN-SGCCDCGDDEAWRTPLFC   87-152
CaUbr1    CAKPFQPGDPVYRCDECGFDSTCVLCASCFNKEDHLNHNVTVYTSSGTSGGICDCGDPEAFIRPLNC   90-156
MmUbr1    CGKVFKSGETTYSCRDCAIDPTCVLCMDCFQSSVHTNHRYKMHTSTG--GGFCDCGDTEAWKTGPFC   99-163
                             *                              * *  *
```

ClpS/N-domain

```
             T2(D251N,H245Y)
                 N  Y
SpUbr11   FRVMLWNDEVHTFDAVVGSVLEALDSSNTAFGLEVAQRVDSIGRFAVA------TSASVHEAIRIANAISKEN-LAVNV 244-315
ScUbr1    YTVIIYNDEYHNYSQATTALRQGVP--DNVHIDLLTSRIDGEGRAMLK------CSQDLSSVLGGFFAVQTNG-LSATL 311-380
FvUbl1    FALILWNDEKHTVQEVQDQVARACRK-SRKLAAENAWETDAIGRSILT------YMPDVKRLLDMAAIMEAIR-VTVTI 236-306
CaUbr1    WALILWNDEFHNLTEAVTAIKAGTGV-DDRNAYKIAEKIDIKGFCVLK------EDDNPQKLIQAKRLVESNG-LVATI 240-310
MmUbr1    YYCVLFNDEHHSYDHVIYSLQRALDC-ELAEAQLHTTAIDKEGRRAVKAGVY-ATCQEAKEDIKSHSENVSQHPLHVEV 226-302
EcClpS    YKVILVNDDYTPMEFVIDVLQKFFSY-DVERATQLMLAVHYQGKAI--CGVFTAEVAETKVAMVNKYARENEHPLLCTL  28-103
CcClpS    YRVLILNDDYTPMEFVVYVLERFFNK-SREDATRIMLHVHQNGVGV--CGVYTYEVAETKVAQVIDSAREHQHPLQCTM  41-116
              ****  * *  *                    *  **                                 *
```

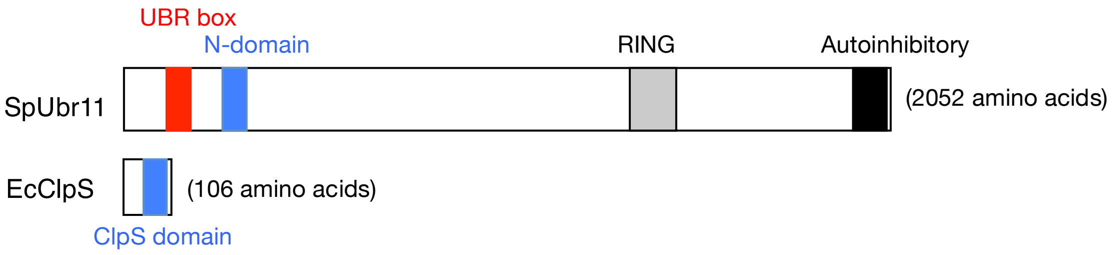

Kitamura Figure S1
