# Supplementary material for: The ClpS-like N-domain is essential for the functioning of Ubr11, an N-recognin in Schizosaccharomyces pombe
Source: Springerplus. 2014 May 20;3:257. doi: 10.1186/2193-1801-3-257 (PMC4447728; doi:10.1186/2193-1801-3-257)
Supplement: Supplementary file 4 — Additional file 4: Table S1: Schizosaccharomyces pombe strains used in this study. (DOCX 73 KB) [file 40064_2014_1547_MOESM4_ESM.docx]

**Table S1.** *Schizosaccharomyces pombe* strains used in this study

| Name | Genotype | Figure |
| --- | --- | --- |
| KSP2 | h^–^ | 3a |
| KSP3 | h^+^ | 3a |
| KSP2261 | ubr11::hphMX6 ura4-D18 leu1-32 h^–^ | 2 |
| KSP2321 | ubr11::ura4^+^ ura4-D18 h^–^ | 3a |
| KSP2522 | leu1^+^-[Pnmt1(^6His^Ubi-Arg^Nd^-^FLAG^GFP)]int h^–^ | S3a |
| KSP2578 | Pk-ubr11-T2(m3) leu1^+^-[Pnmt1(^6His^Ubi-Arg^Nd^-^FLAG^GFP)]int ura4 h^–^ | 1a |
| KSP2586 | leu1^+^-[Pnmt1(^6His^Ubi-Met^Nd^-^FLAG^GFP)]int h^–^ | S3a |
| KSP2610 | leu1^+^-[Pnmt1(^6His^Ubi-Trp^Nd^-^FLAG^GFP)]int h^–^ | S3a, b |
| KSP2694 | ubr11::hphMX6 h^+^ | 3a |
| KSP3009 | isp4::hphMX6 ptr2::ura4^+^ ura4-D18 h^–^ | 3a |
| KSP3121 | ubr11::hphMX6 leu1^+^-[Pnmt1(^6His^Ubi-Arg^Nd^-^FLAG^GFP)]int ura4-D18 | 1a, b, S2a, b |
| KSP3122 | ubr11::hphMX6 leu1^+^-[Pnmt1(^6His^Ubi-Trp^Nd^-^FLAG^GFP)]int ura4-D18 | 1b, S2a |
| KSP3127 | ubr11::kanMX6 h^–^ | 3a |
| KSP3176 | ubr11::hphMX6 leu1^+^-[Pnmt4(^6His^Ubi-ArgRec8C-^FLAG^GFP)]int ura4-D18 h^–^ | 1c |
| KSP3178 | ubr11::hphMX6 leu1^+^-[Pnmt4(^6His^Ubi-TrpRec8C-^FLAG^GFP)]int ura4-D18 h^–^ | 1c |
| KSP3192 | ubr11::kanMX6 ura4 h^–^ | 3b |
| KSP3193 | isp4::kanMX6 ptr2::ura4^+^ ura4-D18 h^–^ | 3a |
